# SUMOylation is not a prerequisite for HSF1’s role in stress protection and transactivation

**DOI:** 10.1038/s41598-025-08735-3

**Published:** 2025-07-05

**Authors:** Miroslav Bardelcik, Oliver Simoncik, Kristina Bednarova, Ondrej Bonczek, Borivoj Vojtesek, Petr Muller

**Affiliations:** 1 Research Centre for Applied Molecular Oncology (RECAMO), Masaryk Memorial Cancer Institute, Zluty kopec 7, Brno, 65653 Czech Republic; 2 Department of Biochemistry, Faculty of Science, Masaryk University, Brno, Czech Republic; 3 Department of Experimental Biology, Faculty of Science, Masaryk University, Brno, Czech Republic; 4 Masaryk Memorial Cancer Institute, Zluty kopec 7, Brno, 65653 Czech Republic

**Keywords:** Heat shock response, HSF1, SUMOylation, Subasumstat, Cancer, Stress, Chaperones, Transcription factors, Sumoylated proteins, Protein folding, Sumoylation

## Abstract

Targeting tumor proteostasis has emerged as a promising strategy in anticancer therapy, particularly through Hsp90 inhibition, which has shown clinical potential. However, the efficacy of Hsp90 inhibitors is limited by the activation of HSF1, a master regulator of the heat shock response (HSR), which mitigates proteotoxic stress by inducing protective chaperones. To address this limitation, we investigated the role of HSF1 SUMOylation in modulating its activity and its impact on Hsp90 inhibitor efficacy. We generated HSF1 mutants with lysine-to-arginine substitutions at five SUMOylation sites and studied their function in H1299 lung carcinoma cells with *HSF1/HSF2* knockout, which lack a functional HSR. Unexpectedly, these mutants retained full transcriptional activity during the early phase of the heat shock response, mimicking the initial stress response of wild-type HSF1. SUMOylation inhibition using Subasumstat also led to altered nuclear stress bodies morphology but did not impair Hsp70 induction or enhance Hsp90 inhibitor cytotoxicity. Our findings reveal that SUMOylation is dispensable for HSF1 activation and transactivation capacity during the early phase of HSR. These results refine our understanding of HSF1 regulation and suggest that alternative strategies targeting HSF1 stability and degradation may enhance the therapeutic efficacy of proteostasis-targeting cancer therapies.

## Introduction

Cellular function and adaptation are intricately regulated by a diverse array of post-translational modifications (PTMs), with SUMOylation emerging as a critical regulator across numerous biological processes^1,2^. Small Ubiquitin-like Modifier (SUMO) proteins are conjugated to target proteins in a highly specific and reversible manner, influencing their localization, stability, and activity^3,4^. This modification is particularly pivotal during cellular stress conditions, where it helps maintain proteostasis and ensures cell survival under adverse environments, such as elevated temperatures^5,6^. A central player in the cellular stress response is Heat Shock Factor 1 (HSF1), a master transcriptional regulator responsible for the induction of molecular chaperones and heat shock proteins (Hsps)^7^. Among these, Hsp70 is one of the most strongly induced targets, serving as a key effector in proteostasis restoration and cellular protection during stress^8^. Upon activation, HSF1 undergoes trimerization, nuclear translocation, and binds to Heat Shock Elements (HSEs) within the promoters of stress-responsive genes, initiating their transcription^9,10^. Notably, this activation process is intricately associated with SUMOylation of HSF1, adding another layer of complexity to the regulation of stress responsive pathways. This process is further complicated by crosstalk with phosphorylation events, wherein specific phosphorylation sites can promote SUMOylation, fine-tuning HSF1’s function during stress responses. However, the precise mechanisms underlying the interplay between phosphorylation and SUMOylation remain poorly understood^11–13^. All SUMO proteins are conjugated to lysine residues through a shared enzymatic machinery^14^. SUMOylation of HSF1 is mediated by a streamlined enzymatic cascade involving an E1-activating enzyme, an E2-conjugating enzyme, and a limited repertoire of E3 ligases; yet it exhibits remarkable specificity in targeting lysine residues on HSF1. Various SUMO ligases have been implicated in this process, suggesting the involvement of complex networks that dynamically regulate HSF1 activity under stress^15–17^. Intriguingly, HSF1 is primarily mono-SUMOylated, although multiple SUMOylation sites have been identified, highlighting the complexity of this regulatory paradigm that governs HSF1 activity^18^. Lysine K298 appears to be a particularly important SUMOylation site and has been shown to undergo modification following stress with the assistance of Hsp27^19^. Many SUMO substrates contain a ΨKxExxSP motif, and phosphorylation near this motif appears to regulate SUMO conjugation on HSF1 as well. In particular, phosphorylation of serine 303 has been identified as a prerequisite for stress-inducible SUMOylation of HSF1, establishing a functional link between phosphorylation and SUMO-mediated control of HSF1 activity^20–22^. The functional implications of HSF1 SUMOylation remain an area of active investigation. Does SUMOylation endow HSF1 with unique functional attributes that are essential for the stress response, or does it serve broader roles, such as promoting protein solubility and preventing aggregation? Recent advances in the development of specific SUMOylation inhibitors provide an unprecedented opportunity to address these questions. Among these, Subasumstat (TAK-981) has emerged as a promising candidate, with potential applications in cancer therapy. TAK-981 inhibited SUMO-activating enzyme (SAE) through a mechanism similar to ML-792, involving the formation of irreversible adducts with SUMO proteins. This process, catalyzed by the enzyme in an ATP-dependent manner, was confirmed via mass spectrometry analysis of SAE inhibition reaction mixtures, revealing species consistent in size with covalent SUMO1-TAK-981 and SUMO2-TAK-981 adducts^23–26^. By employing such inhibitors, researchers can probe the functional relevance of this modification with high precision and decipher the specific contributions of SUMOylation to HSF1-mediated stress responses. In this context, this study aims to elucidate the nuanced interplay between SUMOylation and the cellular stress response, with a specific emphasis on the regulation of HSF1.

## Results

### Investigating the role of specific amino acid sites for sumoylation

**Fig. 1:**
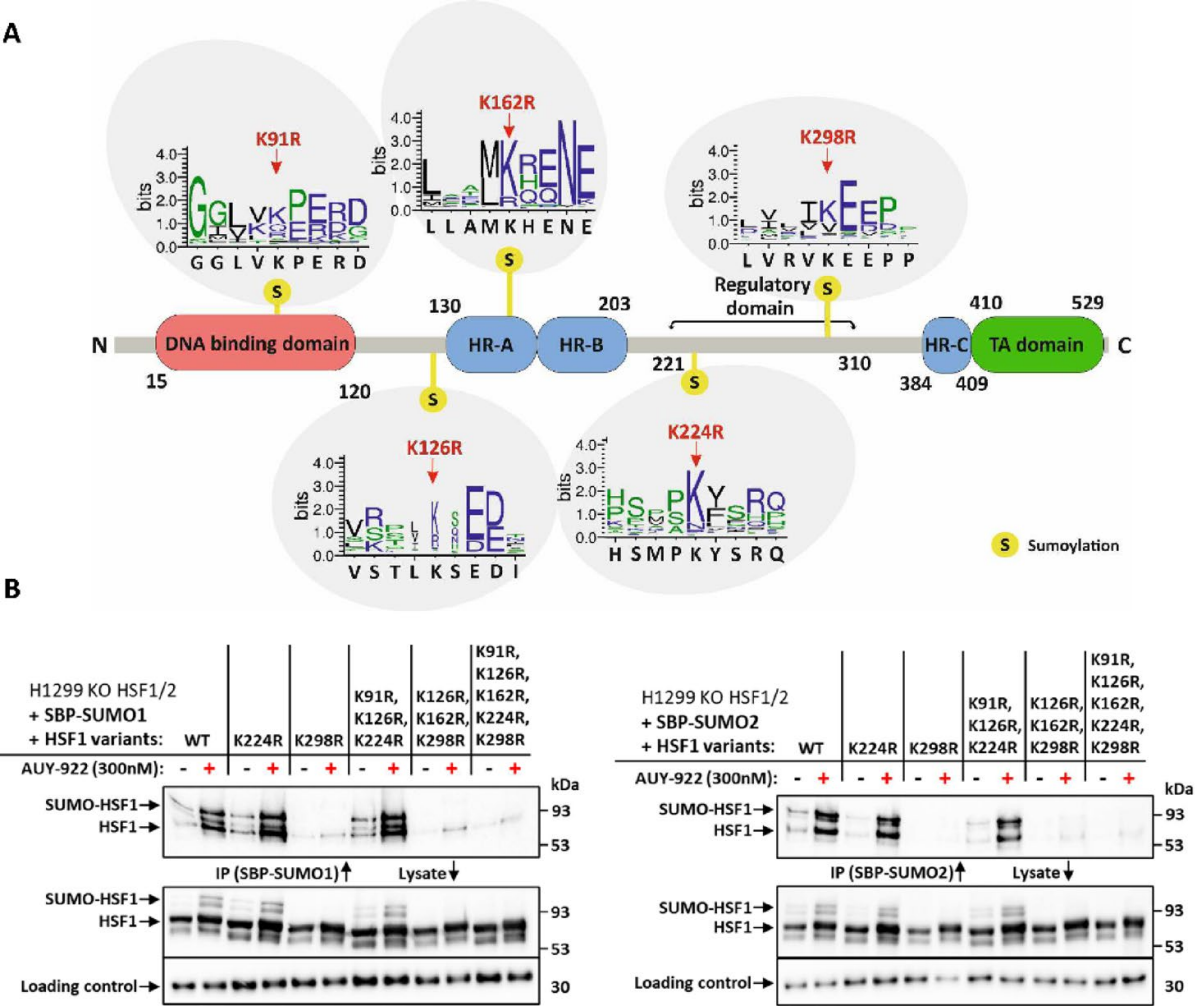
SUMOylation sites and stress-induced SUMO modifications of HSF1. (**A**) Structural organization of HSF1 shows the lysine residues (K91, K126, K162, K224, and K298) that are potentially SUMOylated, along with associated functional domains: DNA-binding domain (amino acids 15–120), hydrophobic repeat regions HR-A and HR-B (130–203), regulatory domain (221–310), HR-C (384–409), and transactivation domain (410–529). A sequence logo illustrates the conserved motifs and sequences surrounding the key SUMOylation sites created using DeepMSA^30,31^. The alignment file is provided in Supplementary Data S1. It highlights the consensus sequences (“ΨKxE,” where Ψ is a large hydrophobic residue), which is critical for SUMO conjugation. (**B**) HSF1 SUMOylation was assessed in H1299 cells stably expressing SBP-tagged SUMO1 (left) and SUMO2 (right), along with WT or mutant HSF1 variants. Cells were treated with AUY-922 (300 nM) for 2 h, followed by SBP pull-down assays to isolate SUMO-conjugated proteins. Western blotting using anti-HSF1 antibody (D3L8, #12972, Cell Signaling) revealed a clear increase in SUMOylated HSF1 upon AUY-922 treatment. Importantly, HSF1 variants carrying the K298R mutation showed no detectable SUMOylation, confirming the critical role of lysine 298 in this modification. PCNA (loading control) was detected in whole cell lysates (#2586, Cell Signaling). The raw data are provided in Raw Data.

**Fig. 2:**
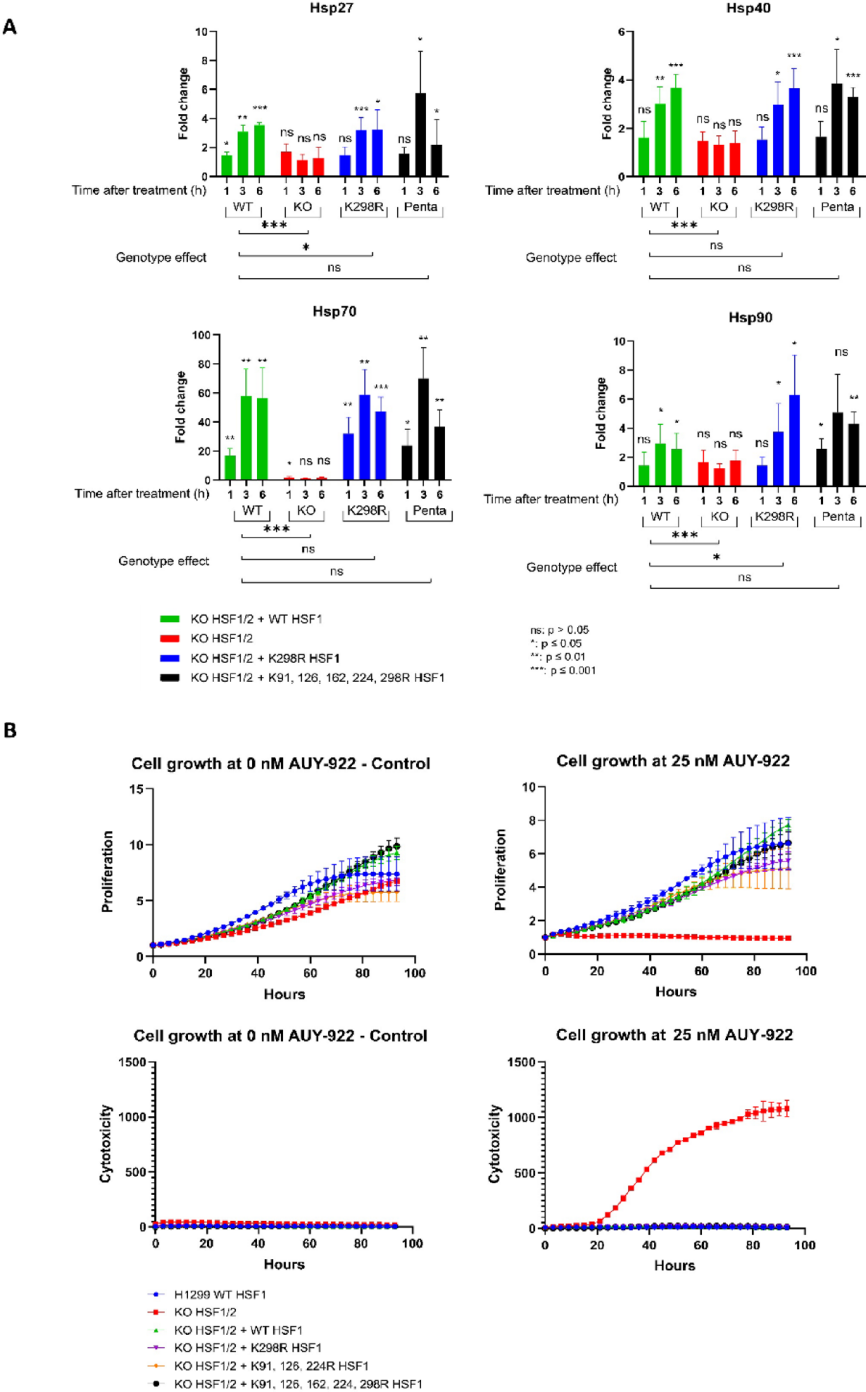
Transcriptional activity and protective ability of mutated HSF1. (**A**) Fold changes in mRNA levels of Hsp27 (*HSPB1*), Hsp40 (*DNAJB1*), Hsp70 (*HSPA1A*), and Hsp90 (*HSP90AA1*) were measured in biological triplicates by one-step RT-qPCR in H1299 cells with *HSF1/HSF2* knockouts, WT HSF1, and HSF1 mutants (K298R and penta mutant: K91R, K126R, K162R, K224R, K298R) transfected into H1299 HSF1/HSF2 -/- cells. Cells were pretreated with the Hsp90 inhibitor AUY-922 (300 nM) for 2 h, after which the compound was removed. Gene expression levels were subsequently measured at 1-, 3-, and 6-hours post-treatment. The results indicate that cells lacking HSF1/HSF2 (-/-) show a significant decrease in chaperone expression, while the K298R mutant and the penta mutant, both of which are unable to undergo SUMOylation, exhibit chaperone expression levels comparable to WT cells. (**B**) The protective capacity of mutated HSF1 variants was evaluated through two metrics: cell proliferation (upper graph) and cytotoxicity (lower graph) using a live-cell imaging system. Cells were seeded and grown in medium containing the Hsp90 inhibitor AUY-922 (25 nM for 93 h) while untreated cells served as controls. Mutated HSF1, which is unable to undergo SUMOylation, protected cells as effectively as WT HSF1 and parental H1299 cell line. In contrast, cells with *HSF1/HSF2* knockout were not protected and exhibited cytotoxicity after 20 h of cultivation with AUY-922.

To elucidate the role of specific lysine residues in HSF1 SUMOylation, we employed site-directed mutagenesis to generate a focused panel of mutants. Candidate lysines were selected based on high SUMOplot prediction scores, partial conservation across species, and their inclusion within or near the ΨKxE SUMO consensus motif. This design was further guided by large-scale proteomic data from Hendriks, I. A. et al.^27^., which identified several putative SUMO acceptor sites in HSF1. Based on these criteria, we generated two single-site mutants (K224R and K298R), two triple mutants (K91R, K126R, K224R and K126R, K162R, K298R), and a comprehensive penta mutant (K91R, K126R, K162R, K224R, K298R) (Fig. 1A). Rather than testing all possible combinations, we prioritized mutants that covered both experimentally identified and computationally predicted SUMOylation sites while minimizing redundancy. Initial functional assays showed that only K298R abolished SUMO conjugation at detectable levels. Given this dominant contribution, additional single or double mutants were not pursued further. All downstream analyses therefore focused on the K298R mutant and the penta mutant, which allowed assessment of both isolated and cumulative effects on SUMOylation. After validation, wild-type (WT) and mutant HSF1 constructs were reintroduced into *HSF1/HSF2* double-knockout H1299 cell lines, generated to eliminate potential compensatory effects between HSF1 and HSF2^28^. Plasmids encoding SBP-tagged SUMO1 or SUMO2 were co-expressed in these cells and used as bait in subsequent pull-down assays. Stable integration and expression of constructs were achieved using the PiggyBac transposase system^29^. To assess the effects of HSF1 mutations on SUMOylation, cell lines were treated with 300 nM Luminespib (AUY-922), and HSF1 SUMOylation was analyzed via pull-down assay followed by Western blotting. Following AUY-922 treatment, we observed an increase in the amount of SUMOylated HSF1 (Fig. 1B). The SBP-SUMO1 and SBP-SUMO2 pull-down assays demonstrated that HSF1 is predominantly mono-SUMOylated by both SUMO1 and SUMO2. In eluates, we observed an upper band corresponding to mono-SUMOylated HSF1 alongside a fraction of non-SUMOylated HSF1. The latter is likely attributable to the trimeric nature of HSF1, where non-SUMOylated subunits co-purify with SUMOylated counterparts. Our results further identified K298 as the primary SUMOylation site. Lysates and eluates from cells expressing the K298R mutant lacked SUMOylated HSF1, confirming that this site is indispensable for the modification. Mutants retaining the intact K298 site continued to undergo SUMOylation, indicating that alterations at other lysines alone were insufficient to block modification. The observation that HSF1 is mono-SUMOylated by both SUMO1 and SUMO2 underscores the importance of these paralogs in regulating HSF1 during proteotoxic stress. Having established the indispensability of K298 for SUMOylation, we subsequently investigated the functional consequences of SUMOylation loss at this site. Initial analyses of HSF1 transcriptional activity in cells expressing the K298R mutant revealed that HSF1-dependent induction of heat shock response genes, including Hsp70, was comparable to that observed in cells expressing wild-type HSF1 following stress exposure (Fig. 2). HSF1 is stress-inducibly SUMOylated at lysine 298, a site recognized as a major SUMO acceptor. Surprisingly, this modification is not required for the induction of the early phase of heat shock response. In fact, previous studies have suggested that SUMOylation at K298 may suppress, rather than enhance, the transactivation capacity of HSF1^11,20^. Our findings align with this model, as HSF1 mutants lacking SUMOylation at K298 retained full transcriptional activity under proteotoxic stress. This indicates that HSF1 can function effectively in the absence of SUMOylation at this site. Thus, the modification appears to be dispensable for acute transcriptional activation. These insights challenge previous assumptions and raise important questions about the broader regulatory significance of K298 SUMOylation, particularly under chronic stress or in disease contexts^22^. Further research is warranted to determine whether SUMOylation at this site influences other processes such as protein stability, interaction dynamics, or long-term adaptation.

### Monitoring transcriptional activity and protective ability of mutated HSF1 dependent on sumoylation

**Fig. 3:**
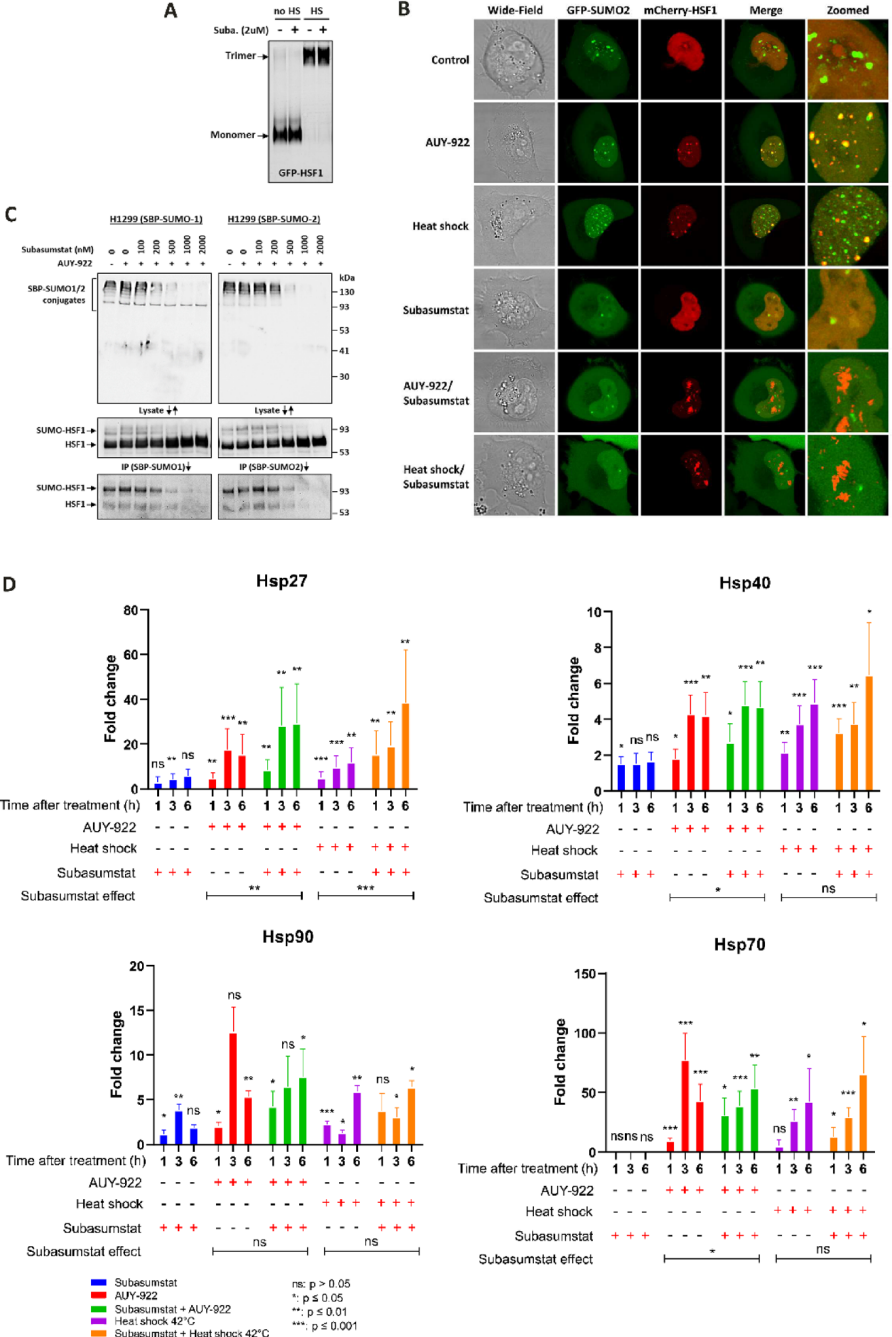

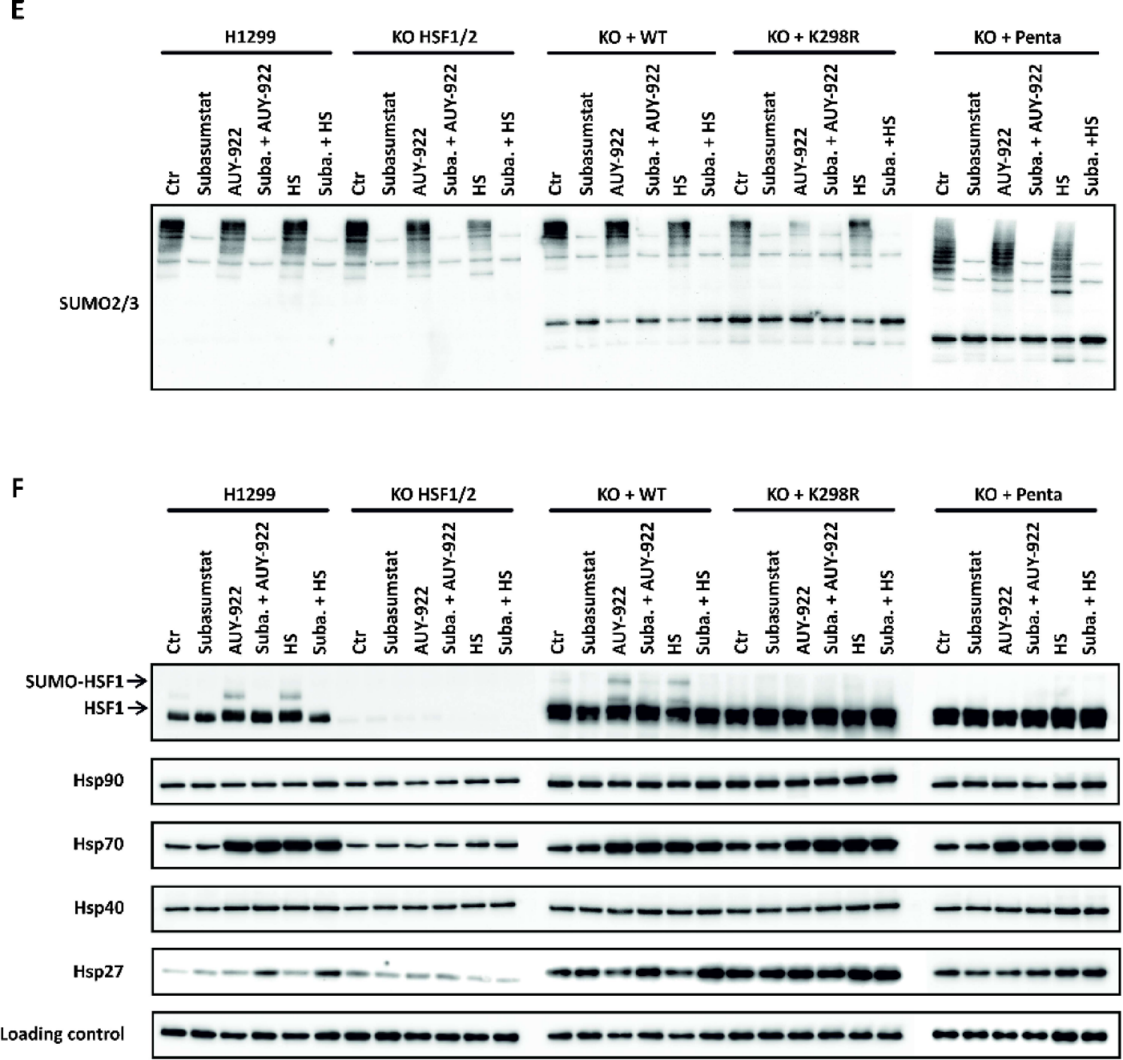
Subasumstat inhibits HSF1 SUMOylation. (**A**) Native gel electrophoresis (HR CNE) of HSF1 trimerization in H1299 *HSF1/HSF2* knockout cells stably expressing GFP-HSF1. Cells were treated with Subasumstat (2 µM, 1 h), followed by heat shock (HS, 42 °C, 1 h). Heat shock-induced trimerization of HSF1 occurs even in the presence of Subasumstat. (**B**) Fluorescence microscopy of H1299 cells stably expressing mCherry-HSF1 and GFP-SUMO2 shows nuclear localization changes in response to AUY-922 (300 nM), HS (42 °C), Subasumstat (2 µM), or their combination following a 2-hour Subasumstat pre-treatment. (**C**) H1299 cells stably expressing SBP-SUMO1 or SBP-SUMO2 were treated with increasing concentrations of Subasumstat (0–2000 nM) for 1 h, followed by exposure to AUY-922 (300 nM) for 2 h. Total SUMOylation was analyzed by Western blot using streptavidin-peroxidase polymer (S2438, Sigma-Aldrich). HSF1 SUMOylation was detected via anti-HSF1 antibody (D3L8, #12972, Cell Signaling). SBP pull-down was performed to specifically evaluate SUMO1/2 conjugation to HSF1, followed by immunodetection of HSF1 in the eluates (IP). The raw data are provided in Raw Data. (**D**) Fold changes in mRNA levels of the chaperones Hsp27 *(HSPB1*), Hsp40 (*DNAJB1*), Hsp70 (*HSPA1A*), and Hsp90 (*HSP90AA1*) were quantified using one-step RT-qPCR. H1299 cells were pretreated with Subasumstat (2 µM) for 2 h, after which AUY-922 (300 nM) was added, or cells were exposed to heat shock at 42 °C for 2 h. Subasumstat remained present throughout the entire experiment. After treatment, AUY-922 was washed out and cells were returned to standard culture conditions (37 °C). Samples were collected at 1-, 3-, and 6-hours post-treatment. (**E**) Western blot of global SUMO2/3-conjugated proteins (anti-SUMO2/3, #4971, Cell Signaling) in parental H1299, *HSF1/HSF2* KO, and KO cells reconstituted with WT HSF1, K298R, or penta mutants. Cells were treated with AUY-922 (300 nM), heat shock (40 °C for 6 h), Subasumstat (2 µM for 8 h), or their combinations. Subasumstat markedly reduced high-molecular-weight SUMO2/3 conjugates, confirming global SUMOylation inhibition. (**F**) Western blot of protein expression levels of HSF1 and chaperones Hsp27 (#13132, Santa Cruz), Hsp40 (#376544, Santa Cruz), Hsp70 (in-house)^32^ Hsp90 (#4877, Cell Signaling) and PCNA – loading control (#2586, Cell Signaling) in parental H1299, HSF1/HSF2 KO, and KO cells reconstituted with WT HSF1, K298R, or penta mutants. Treatments included AUY-922 (300 nM), heat shock (40 °C), Subasumstat (2 µM), or their combinations.

To evaluate the functionality of HSF1 and its dependence on SUMOylation, we monitored both transcriptional activity and the protective capacity of mutated HSF1 in response to cellular stressors. We assessed HSF1 transcriptional activity by analyzing mRNA expression levels of key chaperones using one-step RT-qPCR (primer and probe sequences are detailed in Supplementary Data Table 1). Specifically, we measured the expression of Hsp27 (*HSPB1*), Hsp40 (*DNAJB1*), Hsp70 (*HSPA1A*), and Hsp90 (*HSP90AA1*) in biological triplicates using H1299 cells in which both *HSF1* and *HSF2* were knocked out, and subsequently reconstituted with either WT HSF1, the K298R mutant, or the penta mutant (K91R/K126R/K162R/K224R/K298R). This design allowed us to assess the isolated effect of each HSF1 variant on chaperone gene expression in the absence of interference from endogenous HSF1 or HSF2 (Fig. 2A). The cells were treated with AUY-922 (300 nM) for 2 h, after which the compound was washed out. Chaperone expression was monitored at 1-, 3-, and 6-hours post-treatment to assess HSF1 activity. Fold changes were calculated relative to the corresponding control group at time 0 (untreated). Statistical significance was determined using an unpaired two-tailed *t*-test with a 95% confidence level (*p* < 0.05). In H1299 cells with *HSF1/HSF2* knockouts, the absence of transcriptional activity and the lack of chaperone mRNA induction confirmed the essential role of HSF1 in mediating the stress response. Cells expressing WT HSF1, K298R, or penta mutants exhibited similar transcriptional profiles, with robust induction of chaperone genes following stress. According to a one-way ANOVA testing for genotype effects between WT HSF1 and mutant groups, a statistically significant changes (*p* < 0.05) were observed only for the K298R mutant in the expression of Hsp27 and Hsp90. RT-qPCR results were confirmed by Western blot analysis. Protein levels of Hsp90, Hsp70, Hsp40, and Hsp27 were assessed in parental H1299 cells, *HSF1/HSF2* double-knockout cells, and knockout cells reconstituted with either wild-type or mutant HSF1. This analysis forms part of a broader study in which chaperone protein expression was also evaluated under conditions of SUMOylation inhibition by Subasumstat, as shown in the following section (Fig. 3F). These findings indicate that SUMOylation has minimal impact on chaperone expression at early time points. We further examined the protective capacity of mutated HSF1 variants during treatment with the Hsp90 inhibitor AUY-922 using live-cell imaging to measure cell viability and growth dynamics (Fig. 2B). The parental H1299 cell line, *HSF1/HSF2* double-knockout (*HSF1/HSF2* -/-) cells, and *HSF1/HSF2* -/- cells reconstituted with either wild-type HSF1 or its mutated variants (K298R, the triple mutant K91R/K126R/K224R, and the penta mutant) were analyzed for proliferation and cytotoxicity across a logarithmic concentration range of AUY-922 to model varying degrees of proteotoxic stress (Supplementary Data S2 and S3). Data are presented as mean ± SD from biological triplicates. *HSF1/HSF2* knockout cells showed significantly slower growth and greater sensitivity to stress compared to parental H1299 cells. The transfection of *HSF1/HSF2* -/- cells with either WT or mutated HSF1 genes resulted in a partial restoration of cell growth, but the proliferation rates remained below those of the parental cell line. Importantly, during AUY-922 treatment, cells expressing WT and mutated HSF1 variants exhibited comparable protective effects, with no significant differences in viability or resistance to stress-induced cell death. *HSF1/HSF2* knockout cells began to die within 20 h of treatment, whereas cells expressing WT or mutated HSF1 survived without significant increases in cell death. These results suggest that SUMOylation is not essential for HSF1’s role as a transcription factor or for its ability to protect cells during proteotoxic stress. Regardless of SUMOylation status, HSF1 activation supports cellular survival, maintaining viability and stress resistance comparable to WT HSF1. These findings underscore the robust functionality of HSF1 mutants in orchestrating the heat shock response under adverse conditions.

### Comprehensive inhibition of SUMOylation and its impact on the heat shock response

While the specific loss of SUMOylation at defined HSF1 sites did not significantly affect its transactivation capabilities, we sought to investigate whether global SUMOylation influences the stress response. To achieve this, we utilized Subasumstat a potent inhibitor of the SUMO-activating enzyme (SAE), which blocks SUMOylation not only of HSF1 but also of other proteins that might contribute to the cellular stress response^24,25^. This approach allowed us to evaluate the broader role of SUMOylation in the regulation of HSF1 activity and stress response pathways. First, we evaluated whether SUMOylation inhibition affects HSF1 trimerization, a critical step in its activation. Native gel electrophoresis of H1299 cell lysates expressing GFP-tagged HSF1 showed that heat shock at 42 °C for 1 h induced HSF1 trimerization, even in the presence of Subasumstat (2 µM), indicating that SUMOylation is not required for this process (Fig. 3A). Fluorescence microscopy further supported these findings and revealed additional insights: in cells treated with Subasumstat, heat shock led to a markedly more pronounced accumulation of HSF1 in nuclear stress bodies. Interestingly, these nuclear stress bodies exhibited both larger volumes and irregular shapes compared to controls, suggesting that the absence of SUMOylation (due to Subasumstat treatment) may promote aggregation within these structures (Fig. 3B). Western blot analysis with streptavidin-peroxidase detection confirmed that intracellular proteins are SUMOylated under basal conditions, with increased SUMOylation following Hsp90 inhibition by AUY-922 (Fig. 3C). HSF1 was also found to be SUMOylated under basal conditions, with a slight increase upon stress. Subasumstat effectively blocked these modifications, as further supported by pull-down experiments using SBP-SUMO proteins as baits. Notably, SBP pull-down assays demonstrated that Subasumstat concentrations above 1000 nM significantly reduced HSF1 SUMOylation mediated by both SUMO1 and SUMO2 (Fig. 3C). To assess the functional consequences of global SUMOylation inhibition by Subasumstat, we measured the expression of the chaperones Hsp27 (*HSPB1*), Hsp40 (*DNAJB1*), Hsp70 (*HSPA1A*), and Hsp90 (*HSP90AA1*) using RT-qPCR following treatment with AUY-922 or heat shock at 42 °C. Cells were pretreated with Subasumstat (2 µM) for 2 h, after which AUY-922 (300 nM) was added for an additional 2 h or cells were subjected to heat shock for 2 h. Importantly, Subasumstat remained present throughout the entire experiment. Following treatment, AUY-922 was washed out, and cells were returned to standard culture conditions (37 °C). Gene expression was analyzed at 1-, 3-, and 6-hours post-treatment and is presented as fold change relative to untreated H1299 cells. Data are shown as mean ± SD from biological triplicates. Statistical significance was assessed using unpaired two-tailed t-tests comparing each condition to untreated controls (0 h), with significance levels indicated as *p* < 0.05. To evaluate the effect of Subasumstat under AUY-922 and heat shock conditions, one-way ANOVA was performed and is shown below each graph as Subasumstat effect (Fig. 3D). To confirm SUMOylation inhibition, we assessed global levels of SUMO2/3-conjugated proteins by Western blot in parental H1299 cells, *HSF1/HSF2* double-knockout cells, and knockout cells reconstituted with either wild-type HSF1, the K298R mutant, or the penta mutant (Fig. 3E). Subasumstat treatment (2 µM) markedly reduced high-molecular-weight SUMO2/3 species under both basal and stress conditions, confirming potent inhibition of global SUMOylation. These results validate the functionality of Subasumstat in this context and support its use for probing the role of SUMOylation in stress responses. To validate RT-qPCR findings at the protein level, we analyzed the expression of Hsp90, Hsp70, Hsp40, and Hsp27 by Western blot under the same treatment conditions (6-hour stress or 2-hour Subasumstat pre-treatment followed by 6-hour stress). Chaperone induction after AUY-922 (300 nM) and heat shock (40 °C, 6 h) was evident in cells expressing wild-type or SUMOylation-deficient HSF1 variants, aligning with the transcript-level data. Additionally, we observed a mobility shift of HSF1 upon AUY-922 and heat shock treatment, indicative of its SUMOylation (Fig. 3F). Among these, Hsp70 showed the most robust and consistent induction and was readily detected by Western blot. In contrast, changes in Hsp27, Hsp40, and Hsp90 were weaker and in some cases not detectable under these conditions. Knockout cells exhibited minimal chaperone expression, underscoring the essential role of HSF1. Subasumstat did not substantially reduce chaperone levels, supporting the conclusion that SUMOylation is not essential for acute HSF1-driven transactivation during the early phase of the stress response. Despite the formation of aberrant nuclear stress bodies observed via fluorescence microscopy, the transactivation capacity of HSF1 remained largely unaffected. Neither heat shock nor Hsp90 inhibition caused significant changes in chaperone expression under these conditions. These findings suggest that SUMOylation is not essential for HSF1 activation or the transcriptional regulation of downstream genes during the early stages of the heat shock response. However, prolonged SUMOylation inhibition disrupted global metabolic processes, adversely affecting cell survival, highlighting its broader importance in maintaining cellular homeostasis during extended stress periods.

## Discussion

This study sheds light on the intricate role of SUMOylation in regulating HSF1 activity during proteotoxic stress and its implications for cancer therapy. Our findings reveal that SUMOylation, while not essential for HSF1 activation, may serve a modulatory role in fine-tuning the stress response. To specifically dissect the contribution of HSF1 SUMOylation, we employed H1299 cell lines with knockout of both *HSF1* and *HSF2*. This approach allowed us to eliminate potential compensatory or competitive effects from endogenous HSF1/HSF2 isoforms and isolate the function of wild-type or mutant HSF1 in a controlled genetic background. SUMOylation on individual target proteins and pathways is complex and likely highly context-dependent. A striking observation under stress conditions is the rapid net increase in SUMOylated proteins^33–35^. Specifically, SUMOylation at HSF1 lysine 298 was identified as a primary modification site^27,36^. In addition to its role during acute proteotoxic stress, SUMOylation at K298 may also influence HSF1 stability and long-term cellular outcomes such as cancer cell proliferation. Previous studies have implicated this residue in tumor-related functions of HSF1, suggesting that the loss of SUMOylation could have broader implications beyond stress adaptation^36^. Despite this, the K298R mutant and others that abolish SUMOylation exhibit transcriptional activity comparable to wild-type HSF1. This suggests that SUMOylation does not play a pivotal role in initiating the heat shock response but may influence feedback regulatory mechanisms such as monomer refolding or degradation. Notably, the K298 residue can also be acetylated, highlighting its relevance for additional post-translational modifications. Moreover, this position appears to contribute to the regulation of HSF1 turnover. Furthermore, the study demonstrated that mutations of K298 to either glutamine (Q) or arginine (R) do not affect the formation of nuclear stress bodies (NSBs)^37^. Interestingly, this observation aligns with broader patterns seen for other PTMs, where mutation to arginine does not impair function, yet the modification itself may still play an inhibitory role under specific conditions. For instance, in Ydj1, acetylation-mimicking mutations inhibit function, while mutation to arginine has minimal impact^38^. Identifying cellular contexts that promote elevated SUMOylation of HSF1 may uncover critical insights into its regulatory role and stress adaptation mechanisms. Notably, treatment with Subasumstat (TAK-981), a global inhibitor of SUMOylation, induced the formation of aberrant nuclear stress bodies (NSBs) by HSF1. These NSBs were larger and exhibited irregular shapes compared to those in untreated cells. We hypothesize that the loss of SUMOylation during HSF1 activation may contribute to HSF1 aggregation without significantly affecting its transactivation capacity. This observation suggests that SUMOylation plays a less direct role during stress, potentially preventing protein aggregation and enabling functional protein-protein interactions. Such a role is consistent with studies demonstrating that SUMOylation prevents the protein aggregation, supporting the idea that SUMOylation stabilizes proteins under stress conditions^12,13,39^. Interestingly, a study analyzing the Swiss-Prot protein database identified only 48 human proteins containing the ΨKxExxSP motif conserved between human and mouse orthologs, 71% of which are involved in transcriptional regulation^11^. These findings highlight the motif’s specificity and its enrichment in regulatory proteins. In line with this, our experiments demonstrated that Subasumstat effectively blocked SUMO modifications without significantly affecting early heat shock protein expression or the protective capacity of HSF1. However, while we did not observe potentiation of proteotoxic stress upon SUMOylation inhibition in our system, it is conceivable that SUMOylation plays a more prominent role in other models or during prolonged stress. For example, SUMOylation may stabilize protein networks under stress, a function that could become more evident during chronic stress conditions^19,40–42^. These findings emphasize the dispensability of SUMOylation for HSF1 activation and protective functions while redefining its broader role as a contributor to cellular homeostasis under proteotoxic stress. The maintenance of HSF1 trimerization and nuclear localization despite global SUMOylation inhibition highlights the resilience of non-SUMOylation mechanisms in sustaining HSF1 functionality. However, the formation of aberrant nuclear stress bodies upon SUMOylation inhibition indicates that SUMOylation fine-tunes stress responses by preventing aggregation and supporting proper protein interactions. In conclusion, our results suggest that while SUMOylation is not essential for HSF1 activation, it plays a modulatory role in preventing aggregation and stabilizing protein interactions under stress. Future research should investigate the conditions under which SUMOylation becomes indispensable and explore its therapeutic potential, particularly in combination with proteostasis-targeting agents such as Hsp90 inhibitors. Understanding the long-term impact of SUMOylation on cellular stress adaptation and its interplay with other post-translational modifications will provide further insights into its complex regulatory network. While our current approach uses transient overexpression systems, this may obscure more subtle effects of individual HSF1 site mutations. In future studies, CRISPR/Cas9-mediated introduction of these mutations into the endogenous HSF1 locus could provide additional insights by preserving physiological expression levels and native regulatory context.

## Methods

### DNA construct

All HSF1 mutants were derived from the human HSF1 sequence (NM_005526.4) and cloned into the pDONR221 vector using the Gateway cloning system (Thermo Fisher Scientific Inc., Waltham, MA, USA). SBP-SUMO1 and SBP-SUMO2 constructs were derived from NP_003343.1 and NP_008868.3, respectively, and similarly cloned into the pDONR221 vector. These entry clones were then recombined into expression plasmids using the Gateway LR reaction. For the expression of HSF1 mutants, the PB-EF1a-N-mCherry-TEV-PURO-GWs vector was used, while for SUMO1 and SUMO2, the PB-EF1a-N-SBP-IRES-EmGFP-PURO-GWs vector was employed. Annotated plasmid sequences are provided as supplementary files (S1 – S8 File).

### Cell lines cultivation and treatment

The human non-small cell lung carcinoma (NSCLC) cell line H1299 (ATCC CRL5803) was obtained from the American Type Culture Collection (ATCC, Manassas, VA, USA). Cells were cultured in Dulbecco’s Modified Eagle’s Medium (DMEM) supplemented with 10% fetal bovine serum (FBS), 300 mg/L L-glutamine, and penicillin/streptomycin. Cultures were maintained at 37 °C with 5% CO₂ and grown to approximately 80% confluence before experimental treatments. Stable cell lines expressing fluorescently-tagged HSF1, SBP-SUMO1, or SBP-SUMO2 were generated using the PiggyBac transposon system. Transfected cells were selected with puromycin (2 µg/mL) starting 2 days post-transfection. For stable expression of fluorescently-labeled proteins, individual clones were isolated and sorted using a BD FACSAria III cell sorter.

*HSF1* and *HSF2* knockouts were generated in H1299 cells using CRISPR-Cas9-mediated gene editing, as described previously^32^. Briefly, 5 × 10^5^cells were electroporated with recombinant Cas9 protein and synthetic sgRNA in electroporation buffer using an Amaxa Nucleofector (program X-005). sgRNAs targeted HSF1 (Chr.8: 144308915–144308937, GRCh38) and HSF2 (Chr.6: 122399740–122399762, GRCh38), and knockout was confirmed by sequencing both alleles.

### Cell proliferation and viability

H1299 cells, including the wild-type cell line, *HSF1/HSF2* knockout cell line, and *HSF1/HSF2* knockout cells stably expressing wild-type or mutated HSF1 variants, were seeded at a density of 2500 cells per well in 96-well plates containing growth media and incubated overnight. Cells were then treated with a logarithmic concentration range of AUY-922 for 93 h. Cell proliferation and cytotoxicity were monitored by capturing images every 3 h using the IncuCyte SX1-C2 live-cell imaging system (Sartorius Ltd., Goettingen, Germany). Proliferation was quantified as percent confluence based on phase-contrast images, and data analysis was performed by the IncuCyte image analysis software (Sartorius). The number of dead cells was analyzed using the cell-impermeable red dye, Incucyte Cytotox Dye (Cat.No. 4632), at the concentration recommended by the manufacturer.

### Real-Time PCR

To investigate the fold change in expression of chaperones Hsp27 (*HSPB1*), Hsp40 (*DNAJB1*), Hsp70 (*HSPA1A*), and Hsp90 (*HSP90AA1*), H1299 cells stably expressing either WT or mutated HSF1 were seeded at a density of 2 × 10⁵ cells per well in a 12-well plate and cultured overnight under standard conditions. Cells were pre-treated with Subasumstat (2000 nM) for 2 h, followed by treatment with AUY-922 (300 nM) for an additional 2 h. Following treatment, cells were washed with PBS, and RNA was extracted using the RNeasy Mini Kit (Qiagen, Hilden, Germany). The RNA concentration of each sample was quantified by Nanodrop 2000 (Thermo Fisher Scientific) and samples were diluted to a final concentration of 20 ng/µL with RNase-free water. cDNA synthesis and target gene expression analysis were performed using the OneStep RT-qPCR Kit (Generi Biotech, Trebes, Czech Republic). Real-time RT-qPCR was conducted using a QuantStudio™ 5 system (Applied Biosystems, Life Technologies, Thornton, NSW, Australia) with the following thermal cycling program: reverse transcription at 45 °C for 20 min, followed by 40 cycles of PCR consisting of denaturation at 95 °C for 15 s, annealing at 52 °C for 30 s, and extension at 70 °C for 30 s. The relative expression of target genes was calculated based on Ct values and a calibration curve. Data were normalized to the housekeeping gene *STUB1*, and fold changes were determined relative to untreated control cells. Primer and probe sequences are provided in Supplementary Data Table 1.

### Pull-Down assay

To investigate the interactions between HSF1 mutants and SBP-tagged SUMO1 or SUMO2, 1 × 10^7^H1299 cells stably expressing either WT or mutated HSF1 along with SBP-SUMO1 or SBP-SUMO2 were treated with 300 nM AUY-922 for 2 h. Additionally, to assess global SUMOylation, cells expressing SBP-SUMO1 or SBP-SUMO2 were treated with increasing concentrations of Subasumstat (0–2000 nM) for 1 h, followed by exposure to 300 nM AUY-922 for 2 h. Following treatment, cells were washed with cold phosphate-buffered saline (PBS) containing 20 mM N-Ethylmaleimide (NEM) to inhibit deSUMOylases, scraped into 5 mL of cold PBS containing 20 mM NEM, and pelleted by centrifugation at 1000 ﻿× g for 5 min at 4 °C. Cell pellets were lysed in wash buffer (20 mM HEPES, pH 7.4, 150 mM potassium acetate, 2 mM MgCl_2_) containing 1% Triton X-100, 10 µg/mL avidin, protease inhibitor cocktail (1:100), and 1 mM PMSF. Lysates were sonicated for thorough disruption and centrifuged at 10,000 ﻿× g for 10 min at 4 °C. The resulting supernatants were transferred to new tubes and incubated with 20 µL of high-capacity streptavidin agarose resin beads (Thermo Scientific) for 2 h at 4 °C. Before use, the beads were equilibrated with wash buffer through three consecutive washes, each followed by centrifugation at 8000 ﻿× g for 1 min. Post-incubation with lysates, the beads were washed three times with 1 mL of wash buffer under the same centrifugation conditions. Elution of SBP-tagged proteins was performed using elution buffer (wash buffer supplemented with 1 mM biotin) for 5 min at room temperature. The beads were pelleted by centrifugation at 8000 ﻿× g for 1 min, and the eluates were transferred to fresh tubes. To account for nonspecific protein binding, parallel experiments were conducted using streptavidin beads pre-blocked with 1 mM biotin. Eluted proteins were separated by SDS-PAGE, transferred to membranes, and probed using monoclonal rabbit anti-HSF1 antibody (D3L8, #12972, Cell Signaling, Danvers, MA, USA) to detect levels of HSF1 copurified with SBP-SUMO1 or SBP-SUMO2. SBP-SUMO1/2 conjugates in cell lysates were detected using Streptavidin-Peroxidase Polymer, Ultrasensitive (S2438, Sigma-Aldrich, St. Louis, MO, USA). PCNA in cell lysates was detected using monoclonal mouse anti-PCNA (#2586, Cell Signaling, Danvers, MA, USA).

### High resolution clear native electrophoresis (HR CNE)

Oligomeric states of cellular GFP-HSF1 were determined by High resolution clear native electrophoresis (HR CNE), originally developed by Wittig et al.^43^. Anode buffer (25 mM imidazole pH 7.0) was placed in the outer chamber, while fresh cathode buffer (50 mM Tricine pH 7.0, 7.5 mM imidazole, 0.05% sodium deoxycholate, 0.05% Triton X-100) was placed in the inner chamber of an electrophoretic apparatus. Cell pellets containing GFP-HSF1 were resuspended in chilled lysis buffer (50 mM imidazole pH 7.0, 150 mM NaCl, 2 mM β-mercaptoethanol, 2 mM MgCl_2_, 1% Triton X-100, protease inhibitor cocktail (1:100), Turbonuclease (1:10000), 1 mM PMSF) and lysed on ice for 30 min. Cell lysates were obtained by centrifugation in a pre-cooled centrifuge (4 °C) for 10 min at 8 000 ﻿× g. Cell lysates were mixed with 2﻿× loading buffer (700 µl cathode buffer, 300 µl glycerol, ponceau S (traces), 2 mM EDTA) and separated on 4–10% polyacrylamide gradient gels, prepared as described by Wittig et al.^44^. Electrophoretic apparatus was held on ice and separation was carried out at constant voltage of 110 V for 2 h. Native gels were subsequently scanned using a Typhoon FLA 9500 fluorescence imager. Uncropped raw data corresponding to Fig. 3A are included in the Supplementary Information file labeled Raw Data.

### Western blot

H1299 parental cells, HSF1/HSF2 double-knockout (KO) cells, and KO cells reconstituted with wild-type (WT) HSF1, the K298R mutant, or the penta mutant (K91R, K126R, K162R, K224R, K298R) were seeded at a density of 1 × 10⁶ cells per 5 cm. On the following day, cells were divided into three treatment groups. The first group was treated with AUY-922 (300 nM) and subjected to heat shock at 40 °C for 6 h. The second group was treated with Subasumstat (2 µM) for 8 h. The third group was pre-treated with Subasumstat (2 µM) for 2 h, followed by combined treatment with AUY-922 (300 nM) or heat shock at 40 °C for an additional 6 h, with Subasumstat present throughout. After treatment, cells were washed twice with ice-cold PBS containing 20 mM N-ethylmaleimide (NEM), scraped into 1 mL of PBS + NEM, and centrifuged at 1000 × g for 5 min at 4 °C. Cell pellets were lysed in ice-cold RIPA buffer supplemented with 20 mM NEM, 1 mM PMSF, and a protease inhibitor cocktail. Lysis was performed on ice for 30 min. Lysates were clarified by centrifugation at 14,000 × g for 5 min at 4 °C. Supernatants were mixed with 4× NuPAGE™ LDS sample buffer containing 10% β-mercaptoethanol and boiled at 95 °C for 5 min. Lysates were quantified using the DC Protein Assay (Bio-Rad) and adjusted to a final concentration of 1 µg/µL. For Western blot analysis, 10 µg of total protein was loaded per lane. PCNA was used as a loading control, and protein input was additionally validated using SuperSignal™ Ruby Protein Stain (see Supplementary Data S3). Proteins were separated by SDS-PAGE and analyzed by Western blot using the following primary antibodies: anti-HSF1 (D3L8, #12972, Cell Signaling), anti-Hsp70 (in-house, mouse monoclonal), anti-Hsp27 (F-4, #13132, Santa Cruz), anti-Hsp40 (A-9, #376544, Santa Cruz), anti-Hsp90 (C45G5, #4877, Cell Signaling), and anti-SUMO2/3 (18H8, #4971, Cell Signaling). Uncropped Western blots with visible membrane edges for the experiments shown in Fig. 3E and F are provided in the Supplementary Information (see Raw Data). Please note that membranes were sectioned prior to incubation with primary antibodies to allow parallel probing for different targets. These blots illustrate samples from the main experimental conditions and were not performed using biological replicates.

## Electronic supplementary material

Below is the link to the electronic supplementary material.

Supplementary Material 1

Supplementary Material 2

Supplementary Material 3

Supplementary Material 4

Supplementary Material 5

Supplementary Material 6

Supplementary Material 7

Supplementary Material 8

Supplementary Material 9

Supplementary Material 10

Supplementary Material 11

Supplementary Material 12

Supplementary Material 13

## Data Availability

All data supporting the findings of this study are included in the main manuscript and its Supplementary Information. Primer and probe sequences are detailed in Supplementary Data Table 1. Values from RT-qPCR and live-cell imaging systems are available from the corresponding author upon reasonable request.
