# Supplementary material for: SUMOylation is not a prerequisite for HSF1’s role in stress protection and transactivation
Source: Sci Rep. 2025 Jul 5;15:24077. doi: 10.1038/s41598-025-08735-3 (PMC12228814; doi:10.1038/s41598-025-08735-3)
Supplement: Supplementary file 9 — Supplementary Material 9 [file 41598_2025_8735_MOESM9_ESM.docx]

**Supplementary Data Table 1**

The specific primers and probes used for detecting mRNA levels in the One-Step RT-qPCR method

| Targeted genes | Forward primer | Revers primer | Internal probe |
| --- | --- | --- | --- |
| HSPB1 (Hsp27) | CCAAGTTTCCTCCTCCCTGT | CGGCAGTCTCATCGGATTTT | CATCCCAGTCACCTTCGAGT |
| DNAJA1 (Hsp40) | CTGTCCCAATTGCCGAGGTA | CTTCCTTCCGTTGCAGCTTT | TCAGTCTGTGTGCATGGAGT |
| HSPA1A (Hsp70) | GAGCACAAGAGGAAGGAGCT | CTCCCTTGGGACCCTGAG | TCATCAGCGGACTGTACCAG |
| HSP90AA1 (Hsp90) | GGGCAACACCTCTACAAGGA | ATCAACTGGGCAATTTCTGC | AGGCTACTGATGCCTGAGGA |
| STUB1 (Chip) | TGATGAGGACGACAGCCAC | TGTCTCGCTTCTTCCTCTTCT | AGGCCAAGCACGACAAGTAC |
