# Supplementary material for: SUMOylation is not a prerequisite for HSF1’s role in stress protection and transactivation
Source: Sci Rep. 2025 Jul 5;15:24077. doi: 10.1038/s41598-025-08735-3 (PMC12228814; doi:10.1038/s41598-025-08735-3)
Supplement: Supplementary file 11 — Supplementary Material 11 [file 41598_2025_8735_MOESM11_ESM.docx]

**Supplementary Data S2**

The protective capacity of mutated HSF1 variants was evaluated in comparison to HSF1/2 knockout (-/-) and wild-type (WT) HSF1 using a live-cell imaging system. This included a proliferation study to monitor cell growth and survival under stress conditions.
