# Supplementary material for: SUMOylation is not a prerequisite for HSF1’s role in stress protection and transactivation
Source: Sci Rep. 2025 Jul 5;15:24077. doi: 10.1038/s41598-025-08735-3 (PMC12228814; doi:10.1038/s41598-025-08735-3)

**Supplementary Data S1**

Multiple sequence alignment of HSF1 orthologs generated using DeepMSA (<https://zhanggroup.org/DeepMSA/>). The alignment served as the basis for generating sequence logos to visualize conserved motifs and residues surrounding predicted SUMOylation sites.


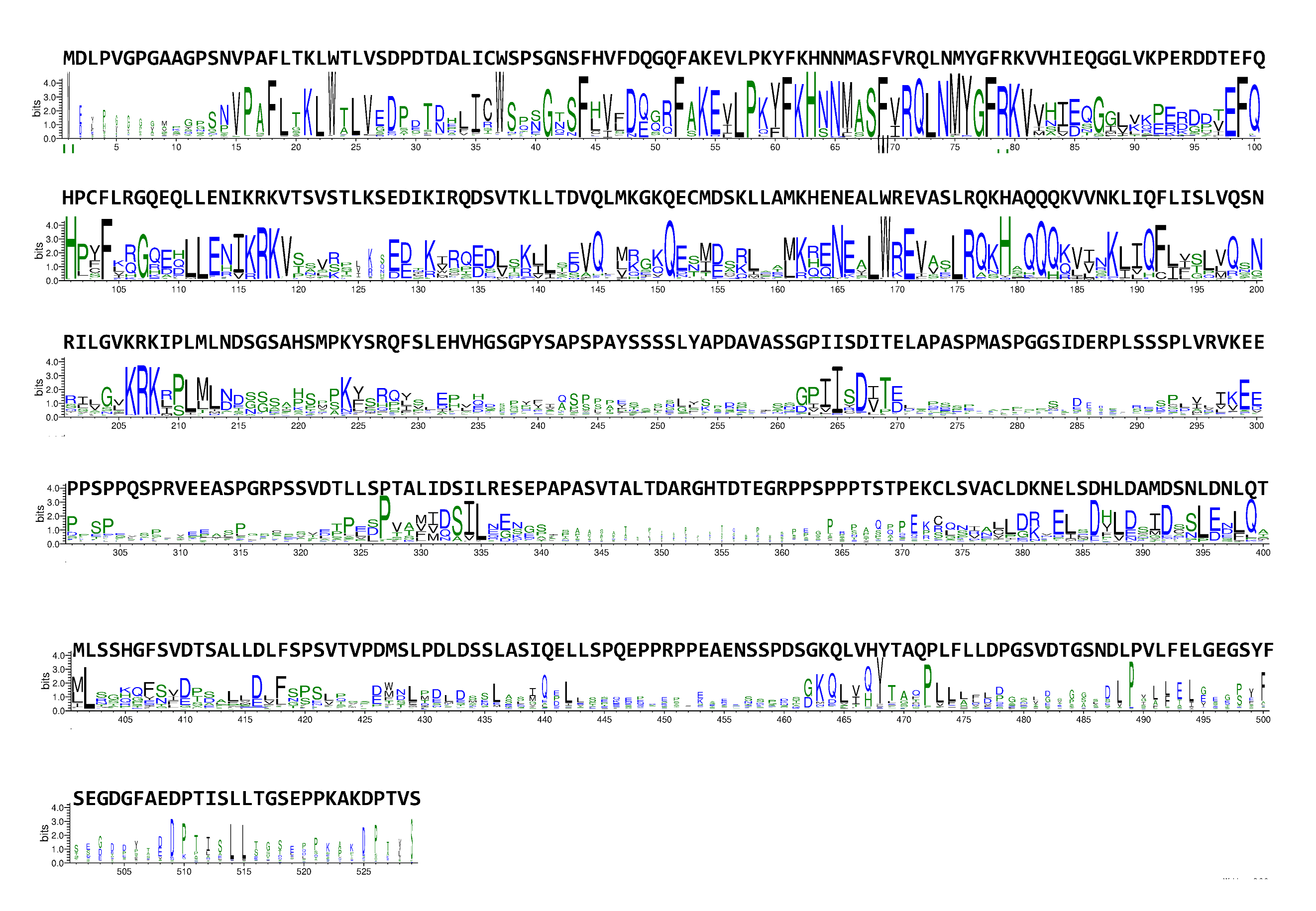

Supplement: Supplementary file 12 — Supplementary Material 12 [file 41598_2025_8735_MOESM12_ESM.docx]
