# Supplementary material for: SUMOylation is not a prerequisite for HSF1’s role in stress protection and transactivation
Source: Sci Rep. 2025 Jul 5;15:24077. doi: 10.1038/s41598-025-08735-3 (PMC12228814; doi:10.1038/s41598-025-08735-3)

**Raw Data to Fig. 1B**

Analysis of HSF1 SUMOylation in H1299 cells stably expressing SBP-tagged SUMO1 (left)


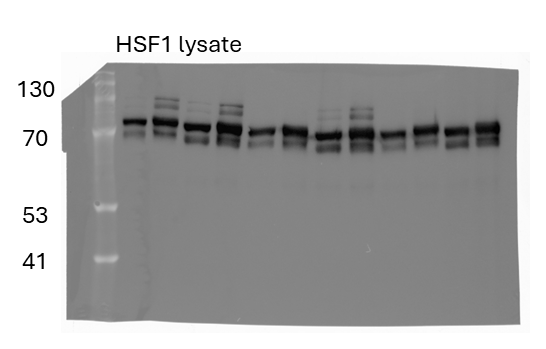

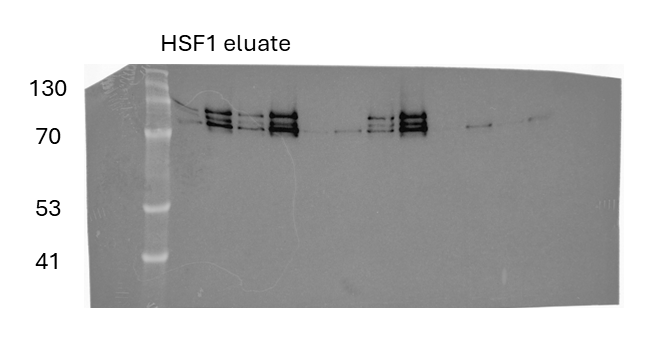


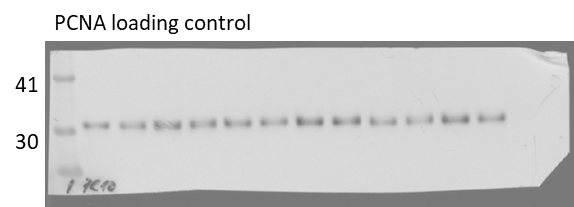


Analysis of HSF1 SUMOylation in H1299 cells stably expressing SBP-tagged SUMO2 (right)


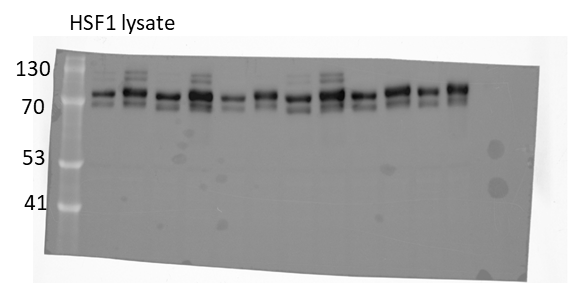

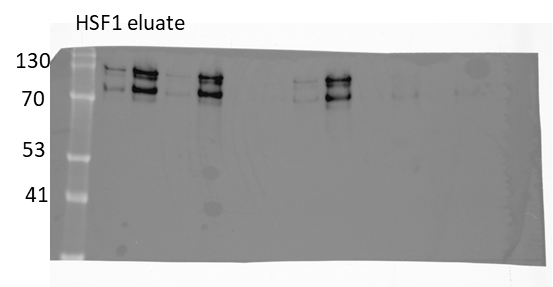


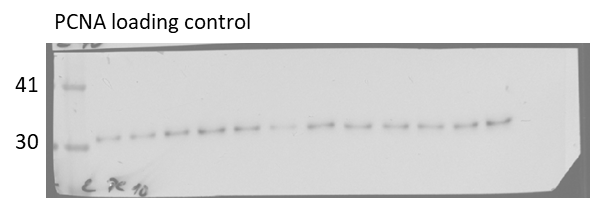


**Raw Data to Fig. 3A**

Native gel electrophoresis (HR CNE) of HSF1 trimerization in H1299 HSF1/HSF2 knockout cells stably expressing GFP-HSF1. Cells were treated with Subasumstat (2 µM, 1 h), followed by heat shock (HS, 42 °C, 1 h). Heat shock-induced trimerization of HSF1 occurs even in the presence of Subasumstat.


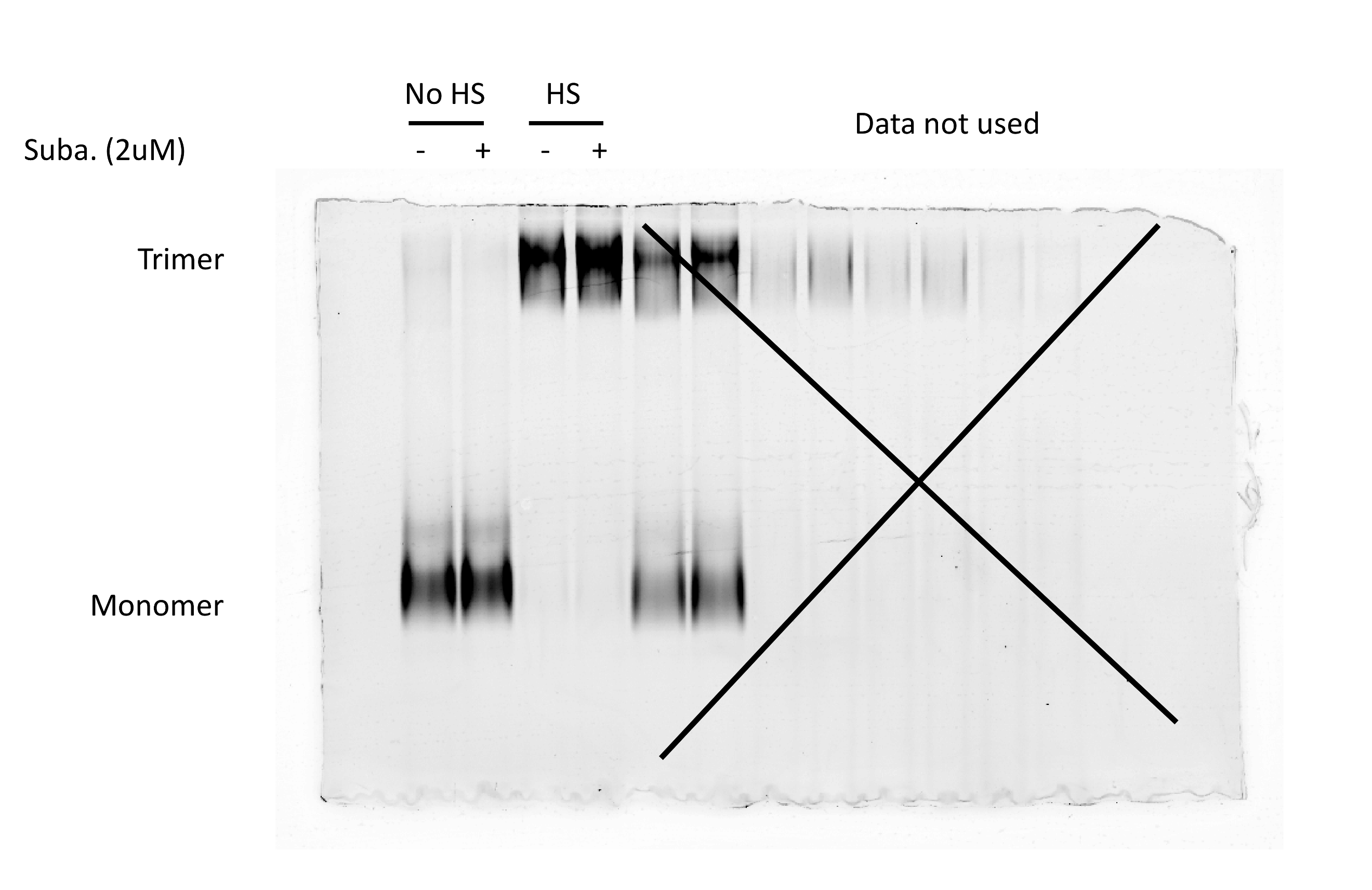


**Raw Data to Fig. 3C**

Western blot analysis with streptavidin-peroxidase polymer detection confirmed that intracellular proteins are SUMOylated under basal conditions, with increased SUMOylation following Hsp90 inhibition by AUY-922.


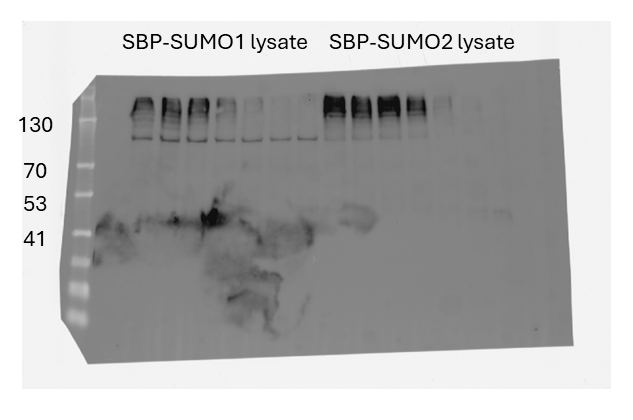


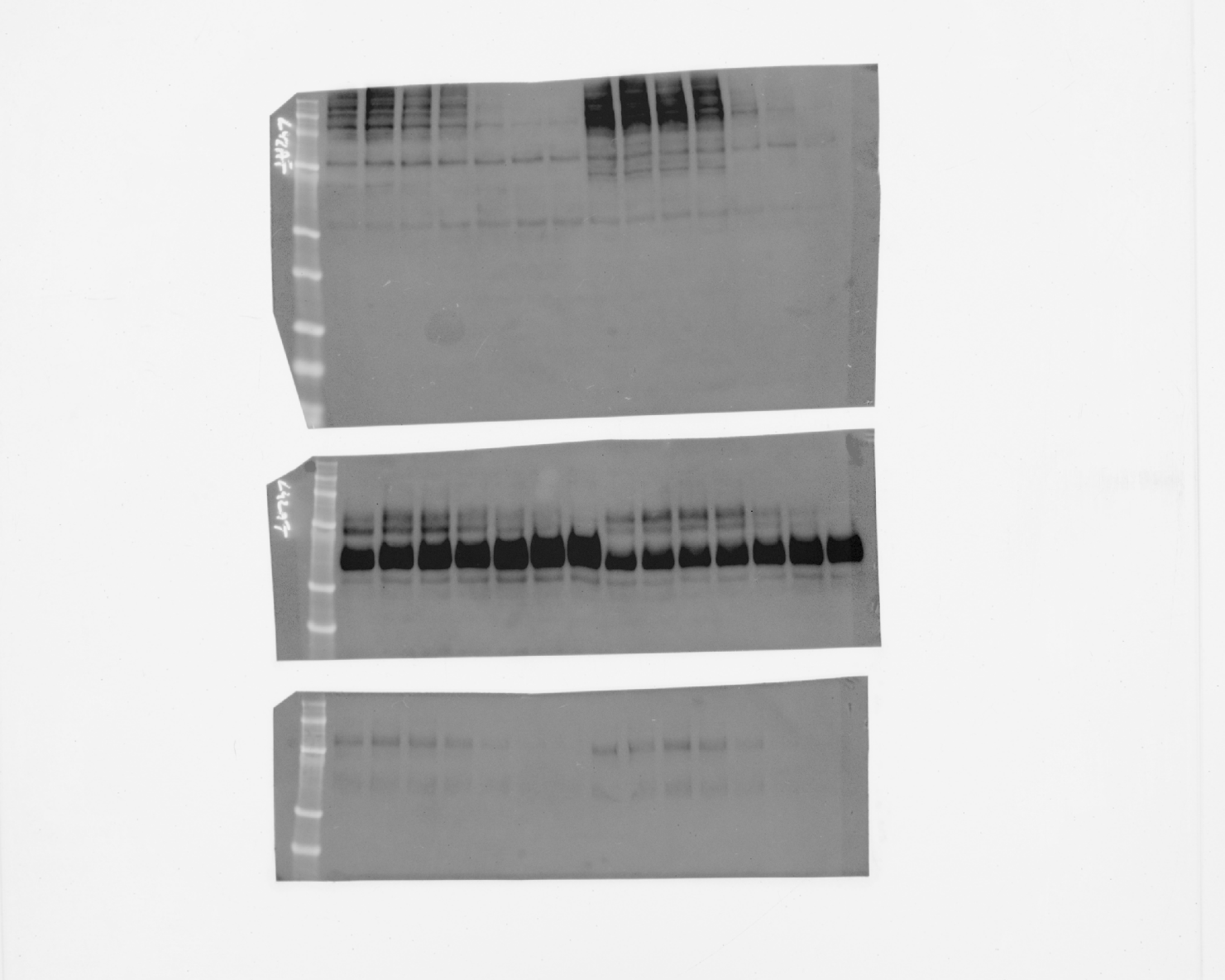

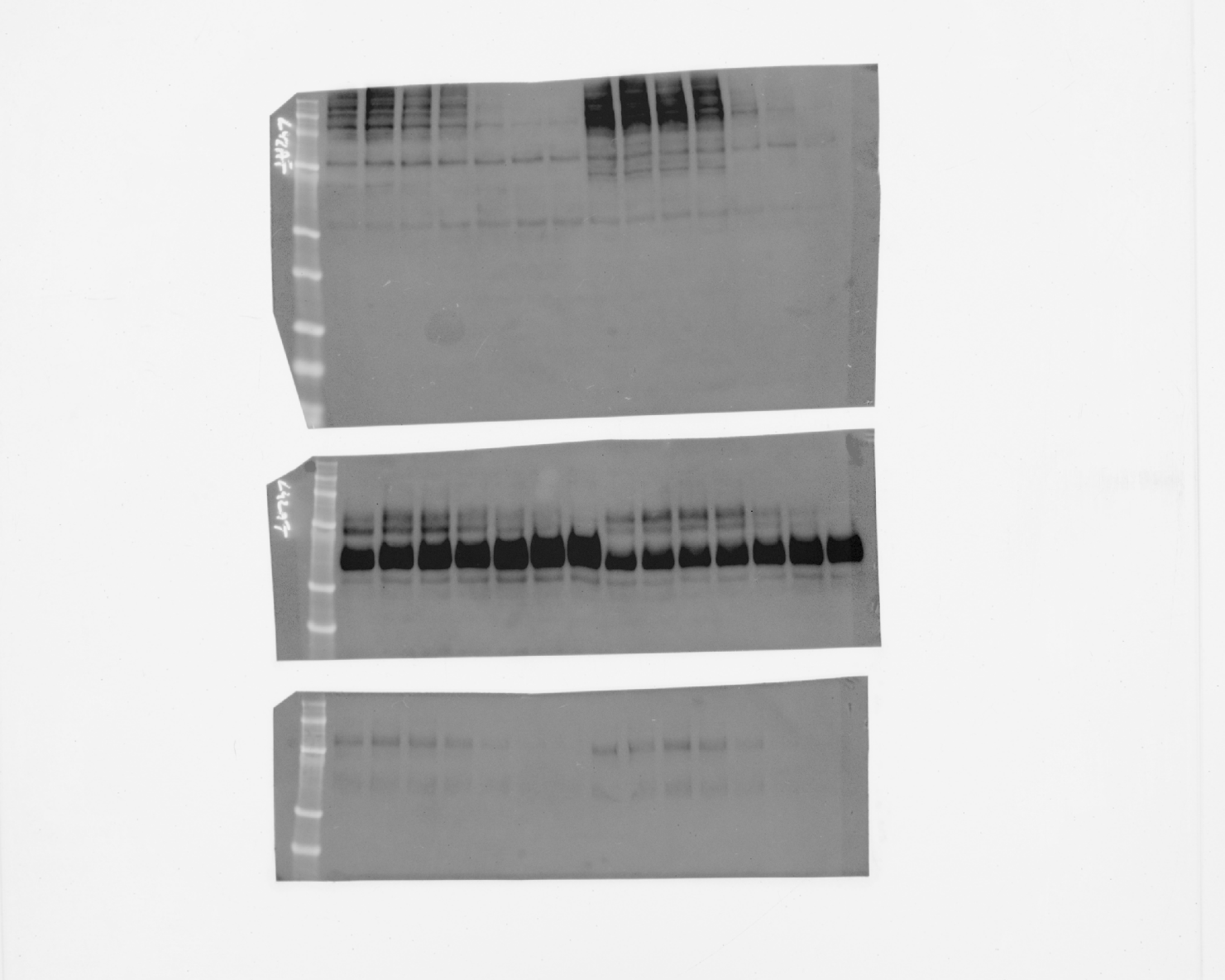


SBP-SUMO2 pull down

SBP-SUMO1 pull down

130

70

53

41

70

53

41

130

SBP-SUMO1 lysate

SBP-SUMO2 lysate

**Raw Data to Fig. 3E**

Western blot of global SUMO2/3-conjugated proteins (anti-SUMO2/3, #4971, Cell Signaling) in parental H1299, HSF1/HSF2 KO, and KO cells reconstituted with WT HSF1, K298R, or penta mutants.


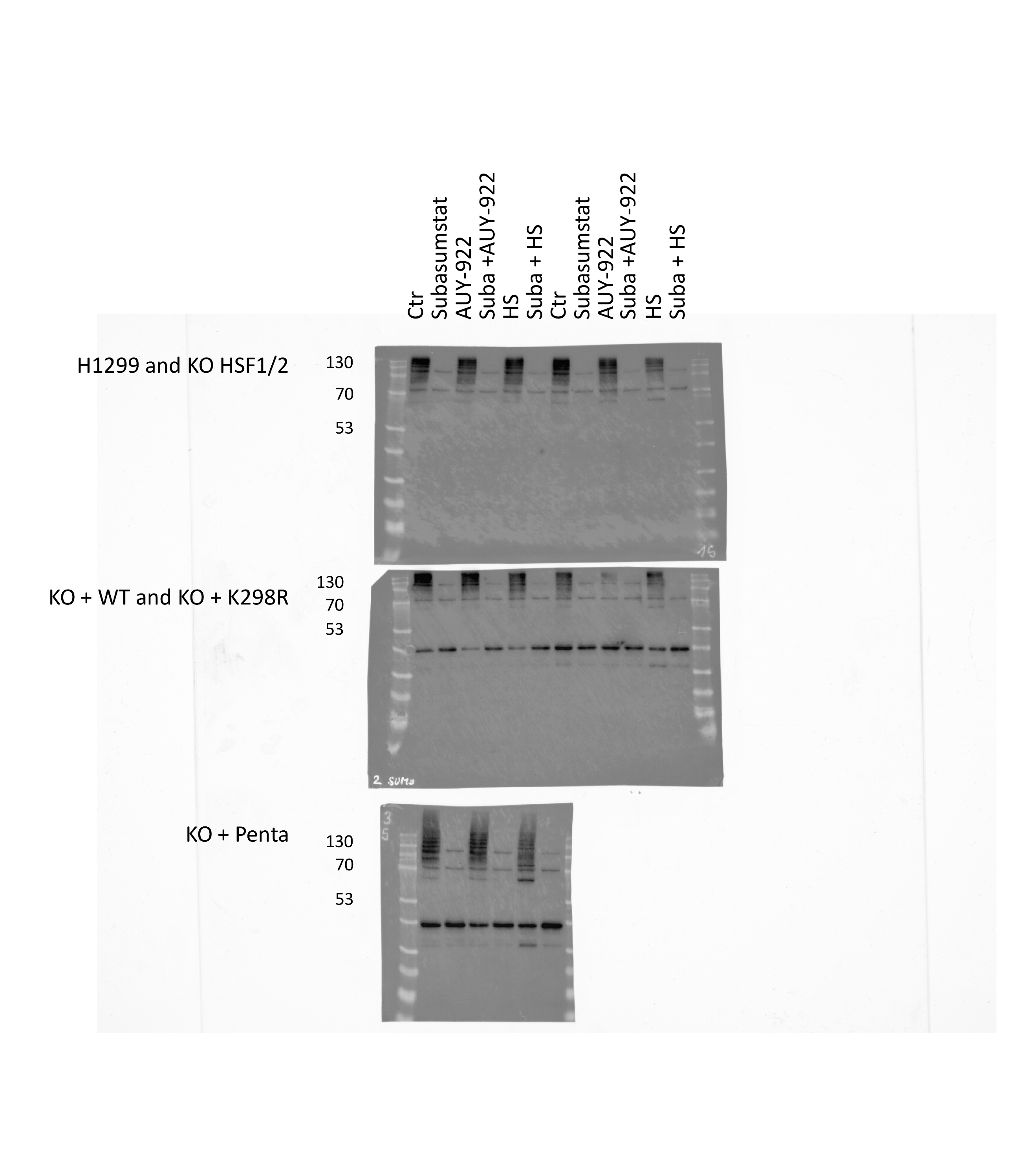


**Raw Data to Fig. 3F**

Western blot of protein expression levels of HSF1 and chaperones Hsp27 (#13132, Santa Cruz), Hsp40 (#376544, Santa Cruz), Hsp70, Hsp90 (#4877, Cell Signaling) and PCNA – loading control (#2586, Cell Signaling) in parental H1299, HSF1/HSF2 KO, and KO cells reconstituted with WT HSF1, K298R, or penta mutants.

1. **HSF1** - from left: (H1299 - Ctr, Subasumstat, AUY-922, Suba. + AUY-922, HS, Suba. + HS) and (KO HSF1/2 - Ctr, Subasumstat, AUY-922, Suba. + AUY-922, HS, Suba. + HS)
2. **HSF1** - from left: (KO + WT - Ctr, Subasumstat, AUY-922, Suba. + AUY-922, HS, Suba. + HS) and (KO + K298R - Ctr, Subasumstat, AUY-922, Suba. + AUY-922, HS, Suba. + HS)
3. **HSF1** - from left: (KO + Penta - Ctr, Subasumstat, AUY-922, Suba. + AUY-922, HS, Suba. + HS)
4. **Hsp70** - from left: (H1299 - Ctr, Subasumstat, AUY-922, Suba. + AUY-922, HS, Suba. + HS) and (KO HSF1/2 - Ctr, Subasumstat, AUY-922, Suba. + AUY-922, HS, Suba. + HS)
5. **Hsp70** - from left: (KO + WT - Ctr, Subasumstat, AUY-922, Suba. + AUY-922, HS, Suba. + HS) and (KO + K298R - Ctr, Subasumstat, AUY-922, Suba. + AUY-922, HS, Suba. + HS)
6. **Hsp70** - from left: (KO + Penta - Ctr, Subasumstat, AUY-922, Suba. + AUY-922, HS, Suba. + HS)
7. **Hsp27** - from left: (H1299 - Ctr, Subasumstat, AUY-922, Suba. + AUY-922, HS, Suba. + HS) and (KO HSF1/2 - Ctr, Subasumstat, AUY-922, Suba. + AUY-922, HS, Suba. + HS)
8. **Hsp27** - from left: (KO + WT - Ctr, Subasumstat, AUY-922, Suba. + AUY-922, HS, Suba. + HS) and (KO + K298R - Ctr, Subasumstat, AUY-922, Suba. + AUY-922, HS, Suba. + HS)
9. **Hsp27** - from left: (KO + Penta - Ctr, Subasumstat, AUY-922, Suba. + AUY-922, HS, Suba. + HS)
10. **PCNA** - from left: (H1299 - Ctr, Subasumstat, AUY-922, Suba. + AUY-922, HS, Suba. + HS) and (KO HSF1/2 - Ctr, Subasumstat, AUY-922, Suba. + AUY-922, HS, Suba. + HS)
11. **PCNA** - from left: (KO + WT - Ctr, Subasumstat, AUY-922, Suba. + AUY-922, HS, Suba. + HS) and (KO + K298R - Ctr, Subasumstat, AUY-922, Suba. + AUY-922, HS, Suba. + HS)
12. **PCNA** - from left: (KO + Penta - Ctr, Subasumstat, AUY-922, Suba. + AUY-922, HS, Suba. + HS)
13. **Hsp90** - from left: (H1299 - Ctr, Subasumstat, AUY-922, Suba. + AUY-922, HS, Suba. + HS) and (KO HSF1/2 - Ctr, Subasumstat, AUY-922, Suba. + AUY-922, HS, Suba. + HS)
14. **Hsp90** - from left: (KO + WT - Ctr, Subasumstat, AUY-922, Suba. + AUY-922, HS, Suba. + HS) and (KO + K298R - Ctr, Subasumstat, AUY-922, Suba. + AUY-922, HS, Suba. + HS)
15. **Hsp90** - from left: (KO + Penta - Ctr, Subasumstat, AUY-922, Suba. + AUY-922, HS, Suba. + HS)
16. **Hsp40** - from left: (H1299 - Ctr, Subasumstat, AUY-922, Suba. + AUY-922, HS, Suba. + HS) and (KO HSF1/2 - Ctr, Subasumstat, AUY-922, Suba. + AUY-922, HS, Suba. + HS)
17. **Hsp40** - from left: (KO + WT - Ctr, Subasumstat, AUY-922, Suba. + AUY-922, HS, Suba. + HS) and (KO + K298R - Ctr, Subasumstat, AUY-922, Suba. + AUY-922, HS, Suba. + HS)
18. **Hsp40** - from left: (KO + Penta - Ctr, Subasumstat, AUY-922, Suba. + AUY-922, HS, Suba. + HS)


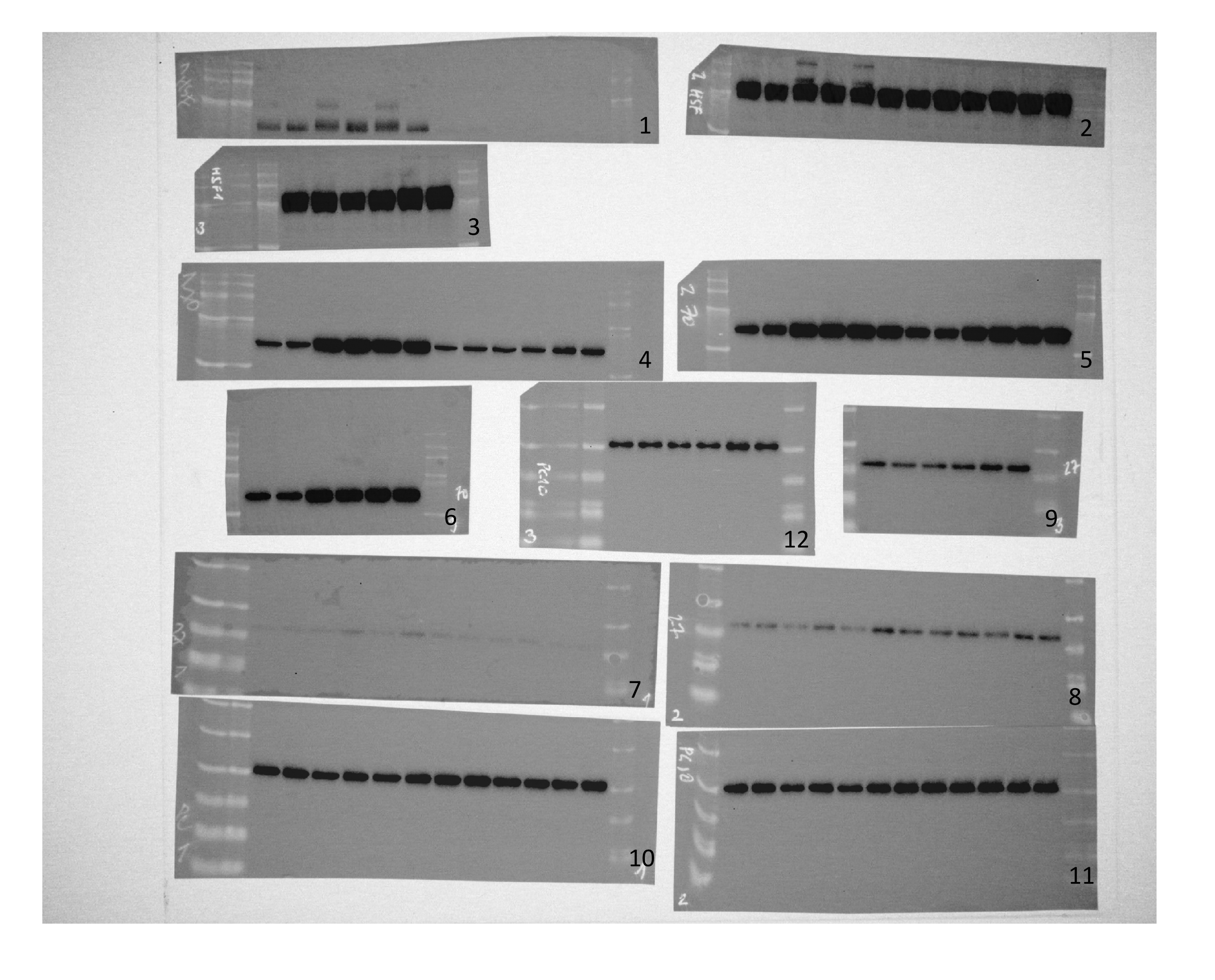

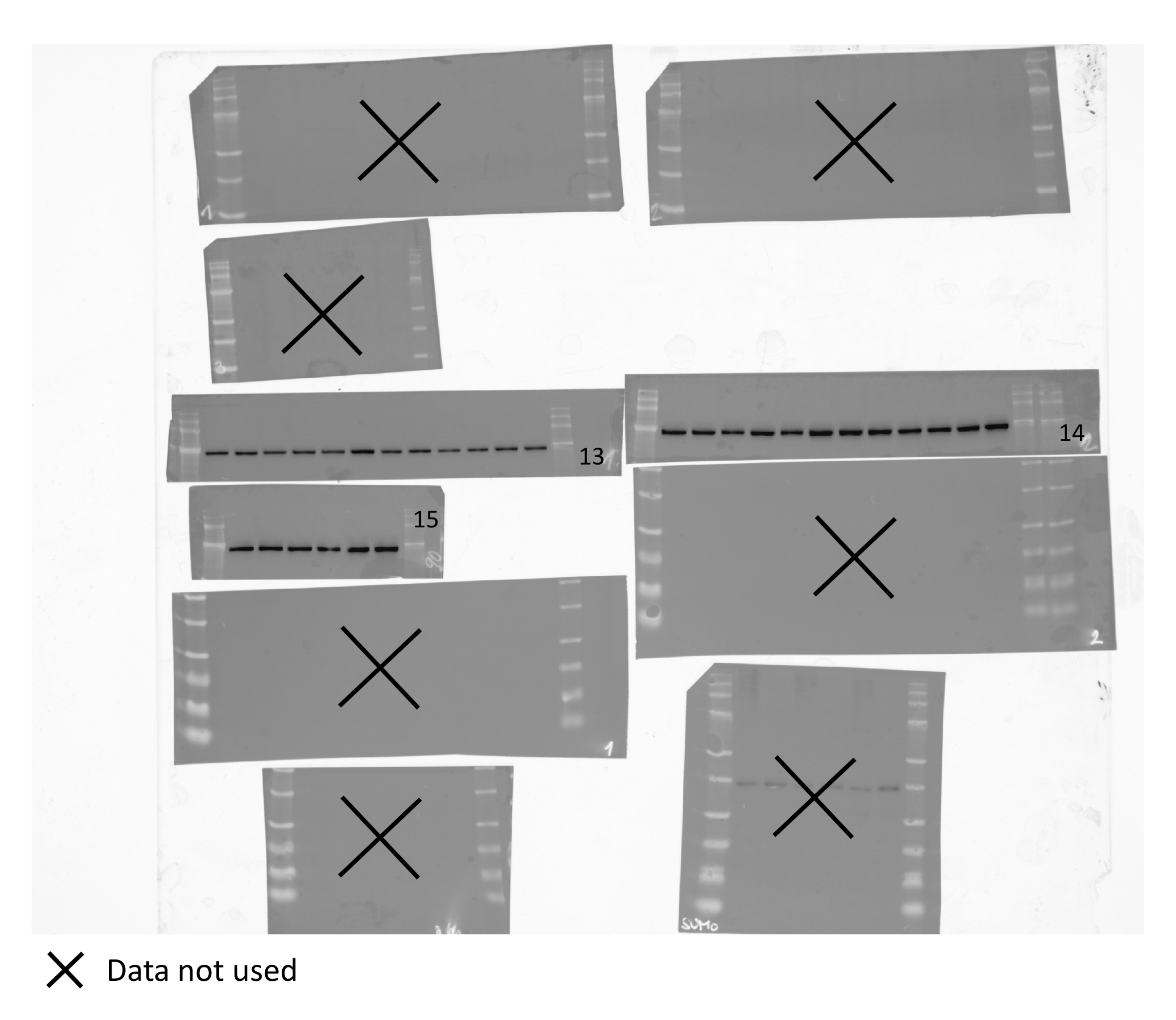


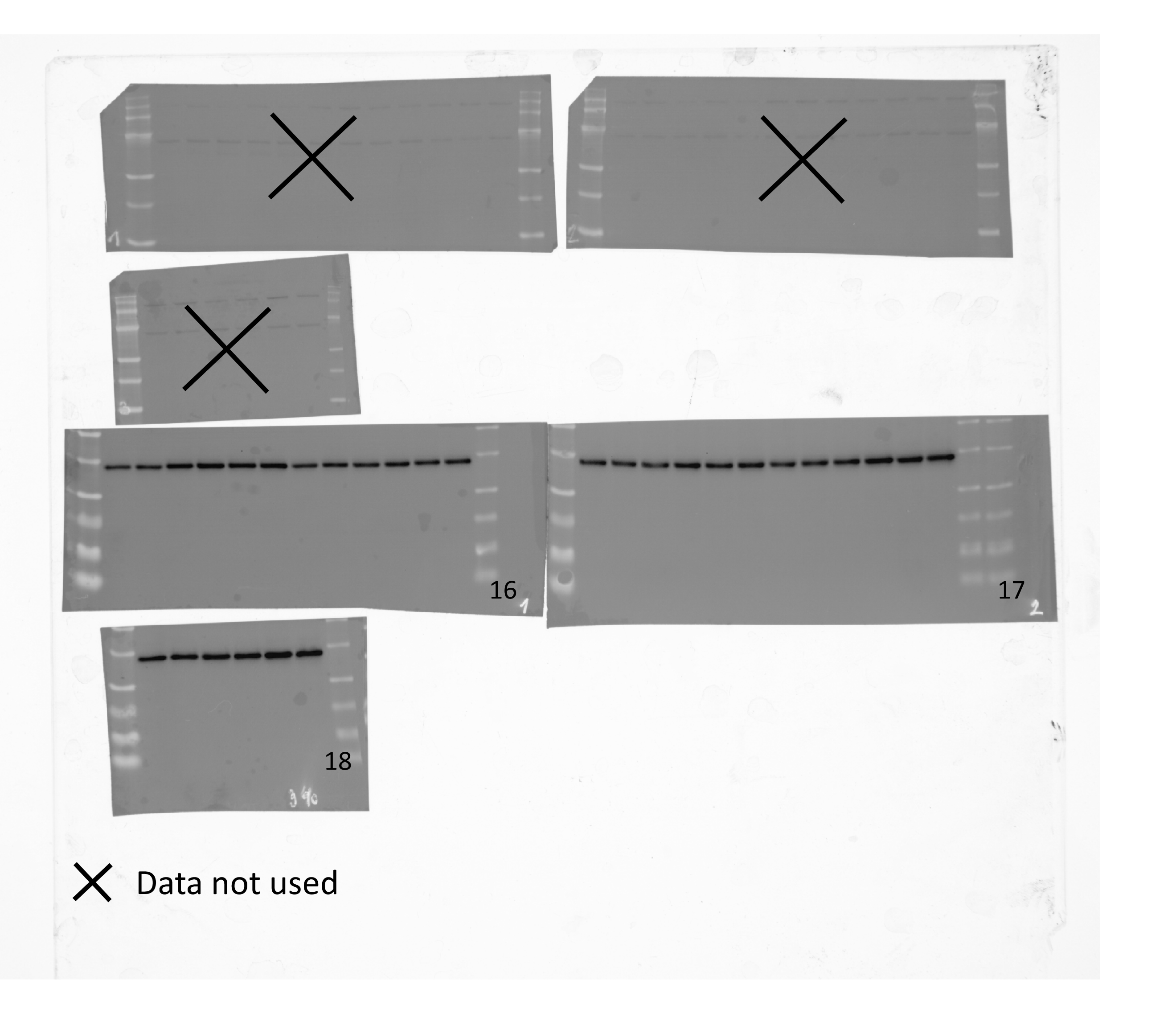

Supplement: Supplementary file 13 — Supplementary Material 13 [file 41598_2025_8735_MOESM13_ESM.docx]
